# A Microfluidic Pump/Valve Inspired by Xylem Embolism and Transpiration in Plants

**DOI:** 10.1371/journal.pone.0050320

**Published:** 2012-11-29

**Authors:** Li Jingmin, Liu Chong, Xu Zheng, Zhang Kaiping, Ke Xue, Wang Liding

**Affiliations:** 1 Key Laboratory for Micro/Nano Technology and System of Liaoning Province, School of Mechanical Engineering, Dalian University of Technology, Dalian, Liaoning Province, People's Republic of China; 2 Key Laboratory for Precision and Non-Traditional Machining Technology of Ministry of Education, School of Mechanical Engineering, Dalian University of Technology, Dalian, Liaoning Province, People's Republic of China; Northeastern University, United States of America

## Abstract

In plants, transpiration draws the water upward from the roots to the leaves. However, this flow can be blocked by air bubbles in the xylem conduits, which is called xylem embolism. In this research, we present the design of a biomimetic microfluidic pump/valve based on water transpiration and xylem embolism. This micropump/valve is mainly composed of three parts: the first is a silicon sheet with an array of slit-like micropores to mimic the stomata in a plant leaf; the second is a piece of agarose gel to mimic the mesophyll cells in the sub-cavities of a stoma; the third is a micro-heater which is used to mimic the xylem embolism and its self-repairing. The solution in the microchannels of a microfluidic chip can be driven by the biomimetic “leaf” composed of the silicon sheet and the agarose gel. The halting and flowing of the solution is controlled by the micro-heater. Results have shown that a steady flow rate of 1.12 µl/min can be obtained by using this micropump/valve. The time interval between the turning on/off of the micro-heater and the halt (or flow) of the fluid is only 2∼3 s. This micropump/valve can be used as a “plug and play” fluid-driven unit. It has the potential to be used in many application fields.

## Introduction

The micropump/valve is the “beating heart” of a microfluidic system [1], [2]. The development of a miniaturized, portable, low cost and easy operation micropump/valve is important [3], [4]. However, present micropumps/valves, have some disadvantages [5], [6], such as requiring a continuous connection with external large equipments, expensive fabrication procedure and unsteady flow rate, which results in the difficulty in integrating these micropumps/valves onto a microfluidic device to obtain a true micro total analysis system (μTAS).

**Figure 1:**
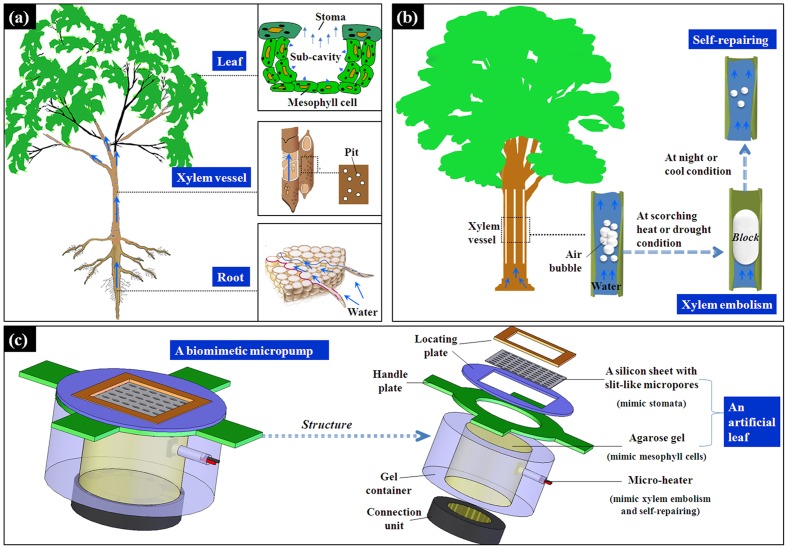
Water transport and xylem embolism in plants and their inspirations in the developing of a biomimetic microfluidic pump. (a) Water transport in plants induced by transpiration through the stomata; (b) xylem embolism induced by cavitation; (c) the structure of the micropump/valve based on water transpiration and xylem embolism.

Transpiration is the loss of water through the slit-like stomata on the leaves, which may generate a water potential gradient in the stem vessels of a plant [7], [8]. The water potential gradient lifts the water upward from the roots, via the xylem vessels and the mesophyll cells, eventually diffusing into the sub-cavities of the stomata (Fig. 1a). Transpiration is a powerful method to drive the fluid [9], [10]. The water can be lifted up to a height of 100 meters with a steady and adjustable flow rate. Because the driving is a passive process, it costs little metabolizable energy of the plant cells [11], [12]. Xylem embolism (Fig. 1b) is mainly caused by cavitation [13]. It readily occurs at scorching heat and drought conditions in which the tension of water generated by the transpiration becomes great enough to separate the air from the water [14], [15]. Embolism can completely block the water transport path in a plant. Xylem embolism can be self-repaired as the atmospheric temperature decreases (especially at night). Solutes can be imported into the xylem conduits via the ray cells or via the bordered pits to redissolve the air-bubbles [16], [17].

In this paper, a biomimetic microfluidic pump is designed based on water transpiration and xylem embolism. Fig. 1c shows the structure of the micropump/valve. We use a silicon sheet with an array of slit-like micropores to mimic the stomata in a leaf. And the agarose gel is used to mimic the mesophyll cells. The silicon sheet and the agarose gel form an artificial “leaf” to drive the fluid. A micro-heater is placed into the agarose gel. Several button cells are used to give the electric power supply to the micro-heater. As the temperature in the agarose is increased by the micro-heater, the air in the agarose gel will expand to block the water transport path, which mimics the formation process of the xylem embolism at scorching heat condition. As the micro-heater is turned off, the temperature in the agarose gel will decrease to open the water transport path, which mimics the self-repairing process of the xylem embolism at night.

Compared with our previous works [18], [19], the micropump/valve presented here mimics the xylem embolism in plants to control the fluid flow. Its structure and bimimetic mechanism are both different. The slit-like micropores which are used to mimics the stomata have high reproducibility in size due to the use of UV-LIGA method in their fabrication. The study of edge effect in a slit-like micropore has been studied by recording the change of the fluorescence density.

## Materials and Methods

### Materials and Solutions

The agarose is purchased from Shanghai Sangon Biological Engineering Technology & Services Co., Ltd. The PMMA plates used to fabricate the micropump/valve are purchased from Asahi Kasei Corporation. The water used in the experiments is deionized water. The micro-heater is a resistance of 1000Ω. The button cell is 1.5 V. Four button cells are used to supply the resistance. The agarose gel is obtained by dissolving the agarose into the deionized water. In this research, we use 8% agarose gel. We firstly measure 8 g agarose and put it into 100 ml deionized water. And then, heat and dissolve the agarose. Then the solution is cooled to room temperature to obtain the agarose gel. The fluorescence solution is obtained by dissolving the fluorescence sodium into the deionized water. The concentration of the solution is about 1 mmol/l.

### Setup

**Figure 2:**
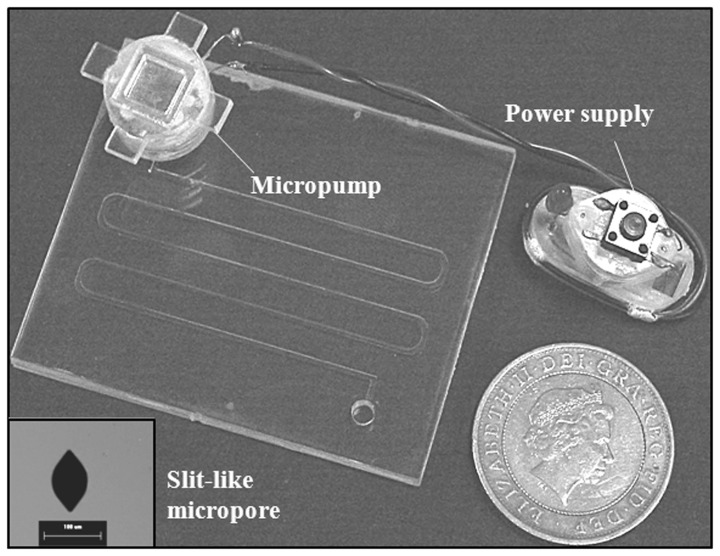
The micropump/valve and the slit-like micropore fabricated by photolithography and wet etching.

A home-made hot embossing machine is used to fabricate the microchip with a serpentine microchannel (shown in Fig. 2). It consists of DC torque motor, lead screw, water cooling block, TEC (Thermal Electric Cooler) blocks, linear encoder and control system. The bonding pressure can be increased to 10000N with a precision of 50N. The temperature can be controlled within room temperature to 150°C. A LIF (Laser Induced Fluorescence) system is used to study the diffusion of water through a slit-like micropore. The LIF system is composed of a biologic microscope (Olympus, IX71) and a CCD (CoolSurf Corporation, 16bit, black/white). The biologic microscope has an argon ion laser of 488 nm. The laser beam can be reflected and focused to a 20 µm-diameter spot. Signal output from the fluorescence chemical sample is detected by a CCD camera. The viewing field is illuminated by using a tungsten filament lamp.

### Micropump/valve Design

As is shown in Fig. 1c, the micropump/valve mainly consists of a silicon sheet with slit-like micropores, agarose gel and a micro-heater. The silicon sheet and the agarose gel form an artificial “leaf” to drive the fluid. The micro-heater mimics the xylem embolism and its self-repairing through increasing or decreasing the temperature in argarose gel. A power supply is used to supply the micro-heater. It has a microcircuit which can control the turning on or turning off of the micro-heater. The array of slit-like micropores on the silicon sheet is fabricated by photolithography and wet etching. The fabricated slit-like micropore is shown in Fig. 2. The length of a micropore is about 98 µm, and the width is 64 µm. The density of the micropore array is 9 micropores per square millimeters. A fabricated micropore has similar shape with a stoma on a leaf.

In additional, the micropump/valve also includes two locating plates, a handle plate, a gel container and a connection unit. The locating plates are used to locate the silicon sheet. The handles in the handle plate is used to move the micropump/valve. They are all fabricated with 2 mm PMMA plates. The gel container is composed of two layers of 2 mm-thick PMMA plates. These two layers are sealed with glue. The connection unit which is hollow and produced with black rubber is used to connect the micropump/valve with the reservoir of a microfluidic chip. The gel container and the connection unit are filled with agarose gel. The locating plates, the handle plate and the gel container are adhered together with glue. The connection unit is inserted into a hole at the bottom of the gel container. Fig. 2 shows the photography of the biomimetic microchip.

### Fabrication of the microfluidic chip

The microchip shown in Fig. 2 has three layers. Its detailed fabrication procedure can be found in our former work [20]. The PMMA substrates and the serpentine microchannel are all fabricated by using CO_2_ laser. The width of the microchannel is about 700 µm, and the depth is about 400 µm. The microchip is 70 mm×50 mm. Three individual layers of the microchip are bonded by using a novel multilayer bonding method which includes “embedded sacrificial layer bonding” and “laser edge welding” [20]. Embedded sacrificial layer bonding is performed by inserting a sacrificial-layer into the reservoirs of a microchip to improve the transfer of bonding pressure among different layers. Laser edge welding is performed by using CO_2_ laser to weld the edge of a bonded multilayer chip. The bonding strength of the microchip is about 320 KPa. No leakage of solution has been observed during experiments.

## Results and Discussion

### Water Potential Generated by the Micropump/valve

This research uses the method reported in our former work to measure the water potential generated by the micropump/valve [19]. A plant water potential meter (TEN-15, Zhejiang TOP Instrument Co., Ltd.) is inserted into a measurement chamber. The chamber is filled with the agarose gel. The water potential generated by the agarose gel will be directly read through the gauge of the water potential meter. It is found that the water potential has reached 72.5 KPa which is large enough to lift water upward to a height of 7 m. We think that the water potential generated by the micropump/valve is composed of two parts. One is the potential generated by the agarose gel. The other is the potential caused by the diffusion of water through the slit-like micropores.

The water potential generated by single agarose gel has been measured. The porous ceramic cup is covered with agarose gel. As the water diffuses from the agarose gel into the air, a vacuum generates within the plastic body tube, which equilibrates with the water potential generated by the agarose gel. Results have shown that the water potential generated by single agarose gel is only about 30∼35 KPa. It can be found that this water potential is only half of that generated by the micropump/valve reported here.

### Halt and Flow of Fluid Controlled by Mimicking Xylem Embolism

**Figure 3:**
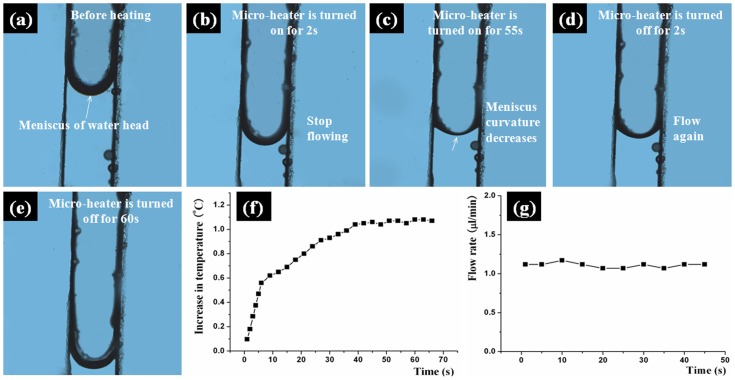
Fluid control and flow rate. (a) to (e) A series of photographs which exhibit the halt and flow of the fluid in a microchannel controlled by mimicking xylem embolism and its self-repairing; (f) the relationship between temperature increase and time; (g) relationship between flow rate and time.

It has been mentioned above that a micro-heater which is placed into the agarose gel is used to mimic xylem embolism to controll the flow of fluid in a microchannel. As the temperature of the agarose gel is increased by the micro-heater, the air in the gel will expand to block the water transport channels, which results in the halting of the fluid flow. As the micro-heater is turned off, the air in the gel shrinks to open the channels, which has mimic the self-repairing process of xylem embolism in a plant. Fig. 3a to e shows a series of photographs which exhibit the halt and flow of the fluid in a microchannel controlled by using this bio-inspired method. Fig. 3a shows the fluid flow before the micro-heater is turned on. Fig. 3b shows the fluid as the micro-heater is turned on for 2 s. The fluid flow has stopped. Fig. 3c shows the meniscus of the water head as the micro-heater is turned on for 55 s. Fig. 3d shows the fluid flow after the micro-heater is turned off for 2 s. The fluid flow has been restarted. Fig. 3e shows the fluid flow after the micro-heater is turned off for 60 s.

To evaluate the performance of this control method, we define closing time and opening time. The closing time is the length of time from the micro-heater being turned on to the fluid flow being stopped. The opening time is the length of time from the micro-heater being turned off to the meniscus of the water head changing obviously which means the transpiration has restarted. It has been found that the closing time and the opening time of this control method are both within 2∼3 s. We have calculated the temperature change of the agarose gel within 2∼3 s. It has been found that the temperature only can be increased or reduced 0.2∼0.3°C within this time. Hence, the response time of this controlling method is very short.

As the turning on state of the micro-heater is retained, the temperature of the agarose gel will increase. However, it has been found that the temperature will keep steady after being increased to 1.0∼1.2°C. Fig. 3f shows the temperature-time profile. The data below 0.5°C are obtained by calculation (by using joule law and the second law of thermodynamics). The data above 0.5°C are measured by a temperature sensor (DS18820, DALLAS). We think that the steady state of the temperature exhibit the balance between heating and dispersing. As the temperature of the agarose gel rises 1.0∼1.2°C, the curvature of the meniscus of water head decreases. But backflow of the fluid is not observed.

### Flow Rate

The research uses the method reported in our former work to measure the flow rate [18]. Firstly, a photo mask with many reference lines (the interval between two adjacent lines is 1 mm) is placed near the microchannel. Secondly, the time taken by the water head to travel between two reference lines is recorded to calculate the flow velocity of the water. Finally, the flow rate is calculated by using a equation of 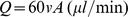, where 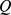 represents the transpiration volume, 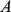 represents the cross-section area of a microchannel, 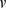 represents the flow velocity. An experiment is performed to test the flow rate of this micropump/valve at 25°C and 50% humidity conditions. We record ten flow rates at different positions of the microchannel. Fig. 3g shows the profile. It can be seen that variation of the flow rate is slight. The maximum value of flow rate is 1.17 µl/min. The minimum value is 1.07 µl/min. The average value is about 1.12 µl/min.

### Effect of Temperature and humidity

We use an air-conditioner to change the ambient temperature and humidity to evaluate the effects of temperature and humidity on flow rate. The temperature is increased from 25°C to 30°C. To simplify the study, the ambient humidity is maintained at 50%. At each temperature degree, we measure three flow rates. It is found that the increase of flow rate is only 0.2 µl/min as the temperature rises from 25°C to 28°C. As the temperature increases to 30°C, the increase of flow rate is 0.3∼0.4 µl/min. It can be found that the micropump/valve can work well at normal temperature, even the temperature has a variation of 2∼3°C. As we study the effects of humidity, the ambient temperature is retained at 26°C. The humidity is increased from 50% to 90% to test the effects of humidity on flow rate. Three flow rates are measured at each humidity level. There are not obvious variations in flow rate as the humidity increases from 50% to 70%. However, an obvious decrease in flow rate is observed as the humidity increases above 80%. As the humidity increases to 90%, the flow rate decreases to about 0.81 µl/min. According to the experimental results, the micropump/valve can work well at a normal humidity condition (below 70%).

## Conclusions

A biomimetic micropump/valve based on transpiration and xylem embolism has been demonstrated in this paper. Results have shown that water potential generated by the micropump/valve is 72.5 KPa which can lift the water upward 7 m. The water potential is not generated by single agarose gel. The diffusion of water through the slit-like micropores can obviously increase the potential due to the edge effects of micropore transpiration. The halt and flow of the fluid has been controlled by mimicking the xylem embolism and its self-repairing behavior. The time interval between the turning on/off of the micro-heater and the halt (or flow) of the fluid is only 2∼3 s. The micropump/valve can work well at normal temperature and humidity conditons with a steady flow rate of 1.0∼1.2 µl/min.
